# The Diagnosis and Treatment of Cystic Tumours in the Scalp

**Published:** 1893-08-19

**Authors:** 


### ROYAL INFIRMARY, EDINBURGH.

### The Diagnosis and Treatment of Cystic Tumours of the Scalp.

It is in the diagnosis of cystic tumours of the scalp that difficulties present themselves. The treatment is a matter of perfect simplicity once the nature of the condition is accurately determined. The conditions likely to be confused with one another are chiefly (1) the ordinary sebaceous cyst or *wen*; (2) the congenital variety of sebaceous cyst or *Dermoid cyst*, and (3) the *Meningocœle*. It is to be borne in mind, however, that tumours, other than cysts, often present physical appearances, which render it not easy to distinguish them from the above. For example, congenital nævi, cirsoid aneurisms, soft sarcomata, or pulsating hæmatomata.

Attention to the following points will aid in the differentiation of the truly cystic tumours:

*(a) Age.*—While the dermoid cyst and meningocœle are for all practical purposes to be regarded as congenital, the common wen as a rule does not appear till well on in life.

*(b) Number and Size.*—The congenital tumours are for the most part single, although frequently more than one dermoid cyst occurs on the same individual. Wens, on the other hand, are almost invariably multiple, and vary greatly in size. On a patient under the care of the writer are to be found between twenty and thirty wens varying in size from a pea to a small cocoanut.

Dermoid cysts, when submitted to treatment, are about the size of a large chestnut, while the meningocœle sometimes attains enormous dimensions.

*(c) Position.*—The ordinary wen, being a retention cyst in a sebaceous gland, obviously may occur in any part of the scalp. It is usually quite free from the skin, unless pressure has been long applied to it, when it may become adherent, and is not attached in any way to the bone or pericranium.

Dermoid cysts are due to a developmental error by which a portion of epiblastic tissue gets infolded and cut off from the rest of the epiblast. They therefore occur most frequently in situations where different embryonal segments fuse in the course of development. For example, in the region of the outer angle of the eye where the fronto-orbital fissure exists, or in the lines of sutures, or branchial clefts. They are free of the skin, but not unfrequently lie so close to the bone as to cause atrophy of it, even to the extent of exposing the dura mater.

The favourite site for meningocœle is in the middle line of the occipital or frontal bones. It may also appear at the root of the nose or in the temporal region. Consisting as it does of a protrusion of the meninges of the brain (dura and arachnoid) through an opening in the skull, the outline of the opening and the pedicle of the swelling may be made out. The skin is usually bluish and tightly stretched over the tumour.

*(d) Physical Character.*—The wen is firm, doughy, and rarely fluctuating; the dermoid cyst is hard or tense and usually fluctuates; both are round or oval. The meningocœle is soft, fluid, and often translucent. It may be reduced partially or entirely into the cranial cavity, but readily reappears, and is rendered tense when the patient cries or coughs.

*Treatment.—Wens.*—The hair over a wen is parted in the direction parallel to the blood vessels, that is, towards the vertex. It need not be cut. The cyst is then transfixed with a sharp knife, the pultaceous contents squeezed out, and the cyst wall seized with forceps and torn or twisted out. It will be found that the most superficial portion of the cyst wall is much thinner than the rest, and if it alone be seized is apt to tear. One blade of the forceps should therefore be pushed well to the bottom of the cyst from the inside, while the other grasps as much as possible outside so as to remove the entire wall without tearing. It is important that no part of the wall be left, otherwise the condition will reproduce itself. The wound is purified and the edges brought together by horsehair stitches, but not until all bleeding has been arrested by the firm pressure of an aseptic pad.

Should the wen be a very large one, or the skin over it be adherent, an elipse of skin may be removed along with the tumour, which had better be dissected out in such cases.

*Dermoid Cysts.*—The wall of these tumours being exceedingly thin, it is found impossible to deal with them in the same way as wens, by evulsion. They must, therefore, be dissected out. It has been already mentioned that they sometimes produce complete atropy of the bone, and come to lie in direct contact with the membranes of the brain. The importance of rigid antiseptic precautions and an aseptic operation is therefore obvious. It often happens that the cyst is ruptured, and the contents escape during the dissection—an accident which renders the operation much more difficult. The best method of procedure in such a case is to freely open the cyst, evacuate its contents, and then stuff the cavity with a strip of lint, so rendering the tumour solid, and greatly facilitating its complete removal. Bleeding is arrested by pressure, and the wound close by horsehair stitches.

*Meningocœle.*—The less these tumours are interfered with by operation the better, as the risk of lighting up an inflammation by the meninges of the brain is very great.

When small in size a pad and elastic bandage may retain the tumour inside the cavity, and give the opening a chance of closing.

When it can be ascertained for certain that no brain tissue is in the tumour, the sac may be exposed and its neck ligatured, but this should only be done if the tumour is growing rapidly and endangering the life of the patient.

